# Association of past dengue fever epidemics with the risk of Zika microcephaly at the population level in Brazil

**DOI:** 10.1038/s41598-020-58407-7

**Published:** 2020-02-04

**Authors:** Marilia Sá Carvalho, Laís Picinini Freitas, Oswaldo Gonçalves Cruz, Patrícia Brasil, Leonardo Soares Bastos

**Affiliations:** 1 Scientific Computation Program, Fundação Oswaldo Cruz, Rio de Janeiro, Brazil; 2 Post-graduation Program in Epidemiology in Public Health, Sergio Arouca National School of Public Health, Fundação Oswaldo Cruz, Rio de Janeiro, Brazil; 3 Acute Febrile Illnesses Laboratory, Evandro Chagas National Institute of Infectious, Fundação Oswaldo Cruz, Rio de Janeiro, Brazil

**Keywords:** Viral infection, Epidemiology

## Abstract

Despite all the research done on the first Zika virus (ZIKV) epidemics, it was only after the Brazilian epidemic that the Congenital Zika Syndrome was described. This was made possible due to the large number of babies born with microcephaly in the Northeast region (NE) in a narrow time. We hypothesize that the fivefold difference in the rate of microcephalic neonates between the NE and other regions is partially an effect of the population prior immunity against Dengue viruses (DENV), that cross-react with ZIKV. In this ecological study, we analysed the interaction between dengue fever epidemics from 2001 to 2014 and the 2015/2016 microcephaly epidemic in 400 microregions in Brazil using random-effects models under a Bayesian approach. The estimated effect of the time lag between the most recent large dengue epidemic (>400/100,000 inhabitants) and the microcephaly epidemic ranged from protection (up to 6 years prior) to an increased risk (from 7 to 12 years). This sustained window of protection, larger than described in previous longitudinal studies, is possibly an effect of herd immunity and of multiple exposures to DENV that could boost immunity.

## Introduction

One of the reasons that made it possible for researchers to associate the microcephaly epidemic in the Northeast (NE) region of Brazil with the Zika virus (ZIKV) was the high rate of dispersion of the virus and the large number of babies born with similar characteristics in the same maternity wards, in the same week, and sometimes even in the same day. This repeatedly happened in different cities of the region in a short time^1^. Despite all the research done on the first epidemics of ZIKV in Yap Islands and later in French Polynesia^2–4^, it was only after the Brazilian epidemic that Congenital Zika Syndrome (CZS) was described^5^. This could be due to the small population in the previously affected areas and to the large number of births concentrated in the same hospitals of the Brazilian Unified Health System (SUS). Even so, a robust analysis using secondary databases estimated a peak of 49.9 microcephaly cases per 10,000 live births in the NE region, whereas for the other regions the peak did not surpass 10 cases per 10,000 live births^6^. So far, microcephaly ratios as high as in the Brazilian NE region have not been described in any other region in the world that had undergone a Zika epidemic^7–9^. Several reasons for this discrepancy were suggested – poor data quality^10–12^, the unreliability of secondary data sources^13,14^, virus genetic variation^15^, socioeconomic factors^16^, dengue immunity profile of the population^17^, and women postponing pregnancy or having abortions^18–20^.

The antigenic similarity between ZIKV and dengue virus (DENV), sharing approximately 54% of their amino acid envelope proteins, results in immunological cross-reactivity^21^. *In vitro* studies have shown that anti-DENV antibodies can both enhance and neutralize ZIKV infection^22–26^. In animal models, mice that received plasma with a low level of anti-DENV antibodies had a higher mortality rate after ZIKV infection than mice that received plasma without antibodies. However, all mice that received plasma with a high level of anti-DENV antibodies survived after ZIKV challenge and presented milder symptoms^22^. Human plasma collected ≤100 days after PCR-confirmed DENV infection binds and cross-neutralizes ZIKV *in vitro*. On the other hand, late-convalescent-phase plasma does not harbour durable, high levels of cross-neutralizing antibodies against ZIKV^27^. However, the understanding of cross-neutralizing antibody responses among individuals with prior DENV exposure, particularly how these responses evolve over time and in various transmission contexts in flavivirus-endemic countries is quite unknown^28^.

In summary, it is known that ZIKV shares structural similarities with other flaviviruses, especially DENV, and co-circulates in dengue-endemic regions. ZIKV and DENV cross-immunity can be either protective or enhancing in experimental models. However, many questions still need to be addressed, particularly by placing *in vitro* and *in vivo* findings in an epidemiological context^29,30^. In this article, we analyse the interaction between dengue fever epidemics from 2001 to 2014 and the 2015/2016 microcephaly epidemic in 400 microregions in Brazil.

## Methods

### Data

This is an ecological study using data gathered by the Health Informatics Department of the Brazilian Ministry of Health (DATASUS). All data are publicly available at the DATASUS website (http://www.datasus.gov.br/DATASUS/index.php/index.php?area=02).

The neonates’ data are collected by the Brazilian Live Births Information System (SINASC), which includes a field to inform the presence of an observed malformation and five fields to classify the condition. Since, at the time, CZS did not have a specific code, we considered as a microcephaly case a live-born with the microcephaly code “Q02” (10^th^ edition of the ICD) in any of the five fields. We compared the number of microcephaly cases between 2015–2016, considered the epidemic period, to the 2014 data, both in the exploratory analysis and the maps.

Dengue data are collected by the Brazilian Information System for Notifiable Diseases (SINAN). We obtained the number of dengue fever cases by municipality and epidemiological week from 2001 to 2014. For the same period, annual population data by municipality estimated by the Brazilian Institute of Geography and Statistics (IBGE), the census bureau, were also obtained.

We excluded the North region (where the Amazon forest is located) due to poorer data quality and incomplete birth records, and the South region, not endemic for dengue fever or any arboviral disease.

Maps were made using QGIS (version 3.6.3)^31^ and layers from IBGE (available at https://downloads.ibge.gov.br/downloads_geociencias.htm), and terrain background by Stamen Design (data by OpenStreetMap).

In accordance with the Brazilian Research Ethics, ethical approval is not mandatory for the use of publicly available datasets.

### Exploratory analysis

**Figure 1:**
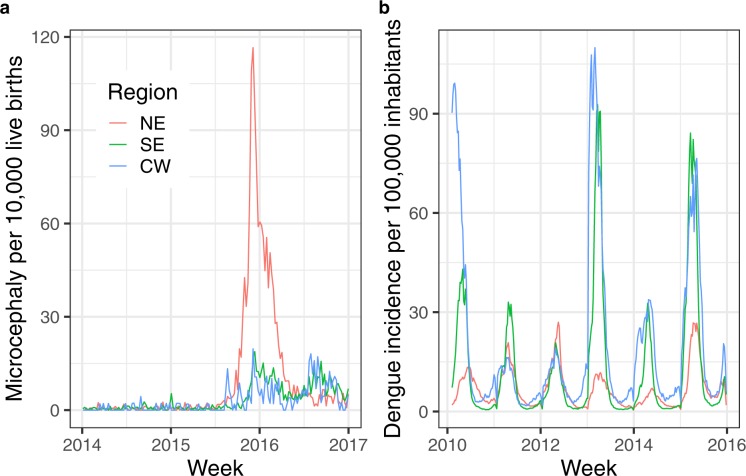
Microcephaly and dengue fever indicators per week per macroregion, Brazil. Frame (**a**) presents the rate of microcephaly cases per 10,000 live-births by week in each region analysed, and frame (**b**) depicts the dengue fever incidence rate per 100,000 inhabitants, in the same time scale.

**Figure 2:**
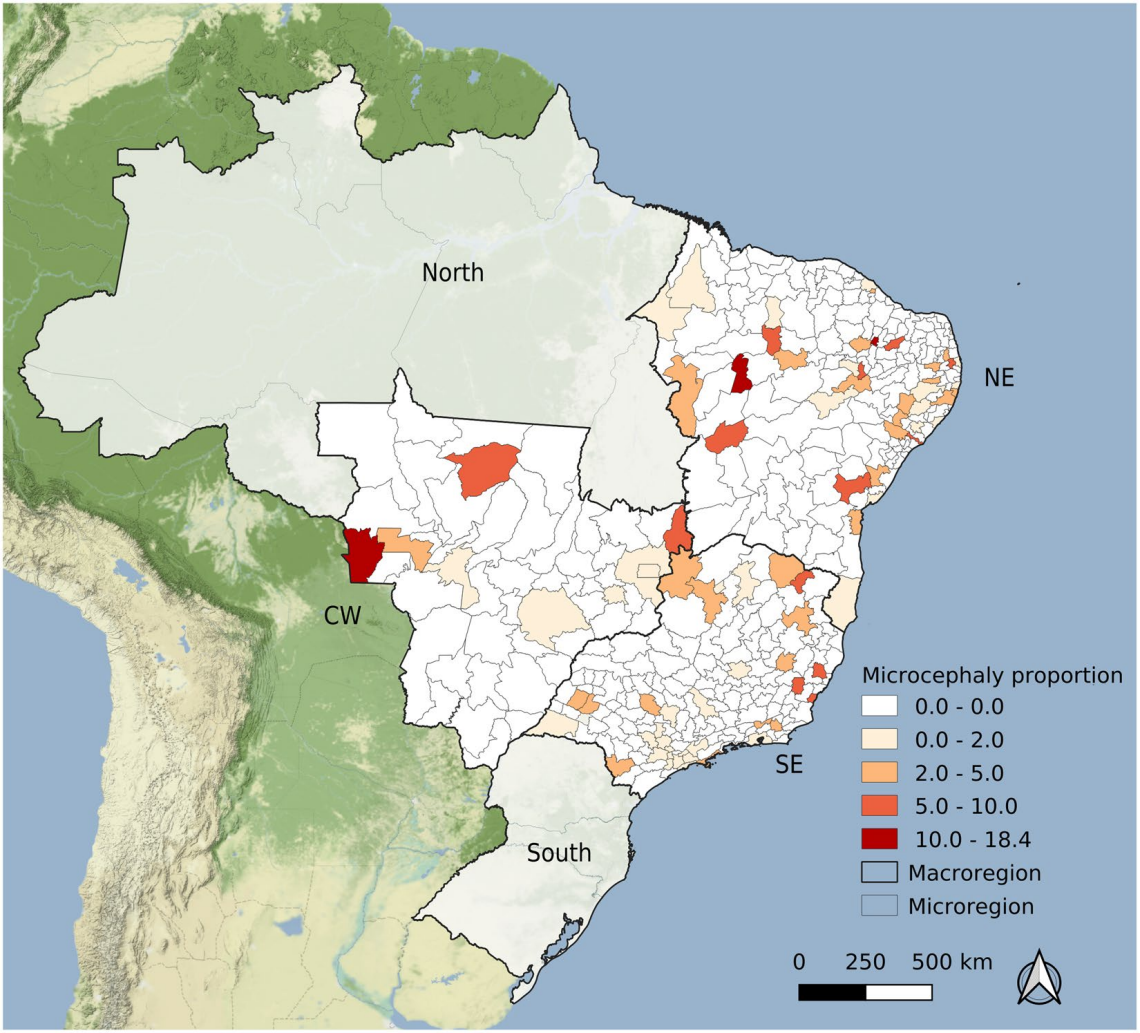
Rate of microcephaly cases per 10,000 live-born infants per microregion, Brazil, 2014. Map created using QGIS version 3.6.3 (QGIS Development Team 2019. QGIS Geographic Information System. Open Source Geospatial Foundation Project. http://qgis.osgeo.org). Map tiles by Stamen Design, under CC BY 3.0. (https://creativecommons.org/licenses/by/3.0/) Data by OpenStreetMap, under CC BY SA.

Live-born data was aggregated by 400 socioeconomically homogeneous microregions, as defined by the census bureau. As dengue fever counts are organized by epidemiological week, the first approach was to use this time scale (Fig. 1a,b). Maps (Fig. 2a,b) present the overall microcephaly rate per 10,000 live-born babies and dengue fever incidence per 100,000 inhabitants by microregion in the three selected Brazilian macroregions: NE, Central-West (CW) and Southeast (SE).

### Models

Using the microcephaly counts as the dependent variable and the total number of live births as offset, we fitted four random-effects models varying the likelihood: Poisson, Negative Binomial, zero-inflated Poisson and zero-inflated Negative Binomial. Two random-effects components were included in the model: the first is a time-varying coefficient for the time since the last big dengue epidemic modelled as a second-order random walk to allow a smooth non-linear behaviour. The second random-effects component was an independent and identically distributed Gaussian random effect by microregion. Vague priors were used and the inference was done under the Bayesian approach using the integrated nested Laplace approximation (INLA)^32^.

To evaluate the time lag between the microcephaly epidemic and a previous dengue fever epidemic, we assessed several rate thresholds (100, 200, 300, 400, 500, and 600 cases per 100,000 inhabitants) and statistical distributions (Supplemental Material). The best fit, according to the Watanabe-Akaike information criterion (WAIC)^33^, was obtained with the zero-inflated Poisson model and 400 cases per 100,000 inhabitants as the threshold of a dengue epidemic.

In brief, the model evaluates the number of microcephaly cases, considering the total number of live births as offset, and dengue fever epidemics with an incidence above 400/100,000 between one and fourteen years prior as an explanatory variable. The model included two random effects: (1) microregion random effects considering both a spatial structured random effect and an unstructured one, allowing for spatial dependence and extra-variability, and (2) spline-like random effects for the time, in years, since the last dengue epidemic in each region.

We used the R software^34^ with the tidyverse library^35^ to organize the data and present the results, and R-INLA^36^ for the analysis. The R code is available in the Supplemental Material.

## Results

The total number of microcephaly cases increased more than 23-fold between 2014 and 2015 in the NE region, and more than tenfold in both the SE and CW regions between 2015 and 2016 (Table 1). The number of live births in 2016 decreased 5% in the NE and CW regions, and 6% in the SE, compared with 2015, following the declaration of public health emergency by the Brazilian Health authorities. In just one week, the number of microcephaly cases in 2015 in the NE region reached 29, more than half of the total number of microcephaly cases from the previous year. The number of microregions with no microcephaly cases decreased first in the NE, the dispersion centre of Zika epidemic, followed by the SE and CW regions.

**Table 1:** Number of microcephaly cases in neonates, number of live births, the maximum number of microcephaly cases in one week, and proportion of microregions with zero microcephaly cases, by year and macroregion, Brazil.

	Year	Northeast	Southeast	Central-West
Microcephaly cases	2014	55	76	12
	2015	1294	283	62
	2016	1071	844	138
Live births	2014	838094	1189848	246450
	2015	844590	1193720	247069
	2016	799760	1123013	233888
Maximum number of microcephaly cases in one week	2014	2	2	1
	2015	29	15	5
	2016	19	10	3
Percentage of microregions with zero microcephaly cases	2014	79.3%	78.8%	82.7%
	2015	22.3%	61.3%	69.2%
	2016	19.7%	37.5%	37.7%

The rate of microcephaly in neonates per 10,000 live births per week by Brazilian macroregions is presented in Fig. 1a. The increase of microcephaly rate beginning at the end of 2015 is observed in all regions. However, in the NE region, by August/2015 the rate had surpassed 30 cases per 10,000 live births, while in other regions it was never higher than 20/10,000 live births. In the SE and CW regions, a second peak occurred at the end of 2016. Comparing the incidence rates of dengue fever (Fig. 1b) in the SE and CW regions, three epidemic years (2010, 2013 and 2015) are present, while in the NE region, in the same period, we observed just a seasonal pattern with no outbreaks.

The map presented in Fig. 2 shows the usual pattern of microcephaly rate among live-born neonates in 2014, before the Zika epidemic. Among the 400 microregions included in the analysis, in 318 (80%) no microcephalic neonate was reported, and the highest rate was due to two babies born in an area with a small population.

**Figure 3:**
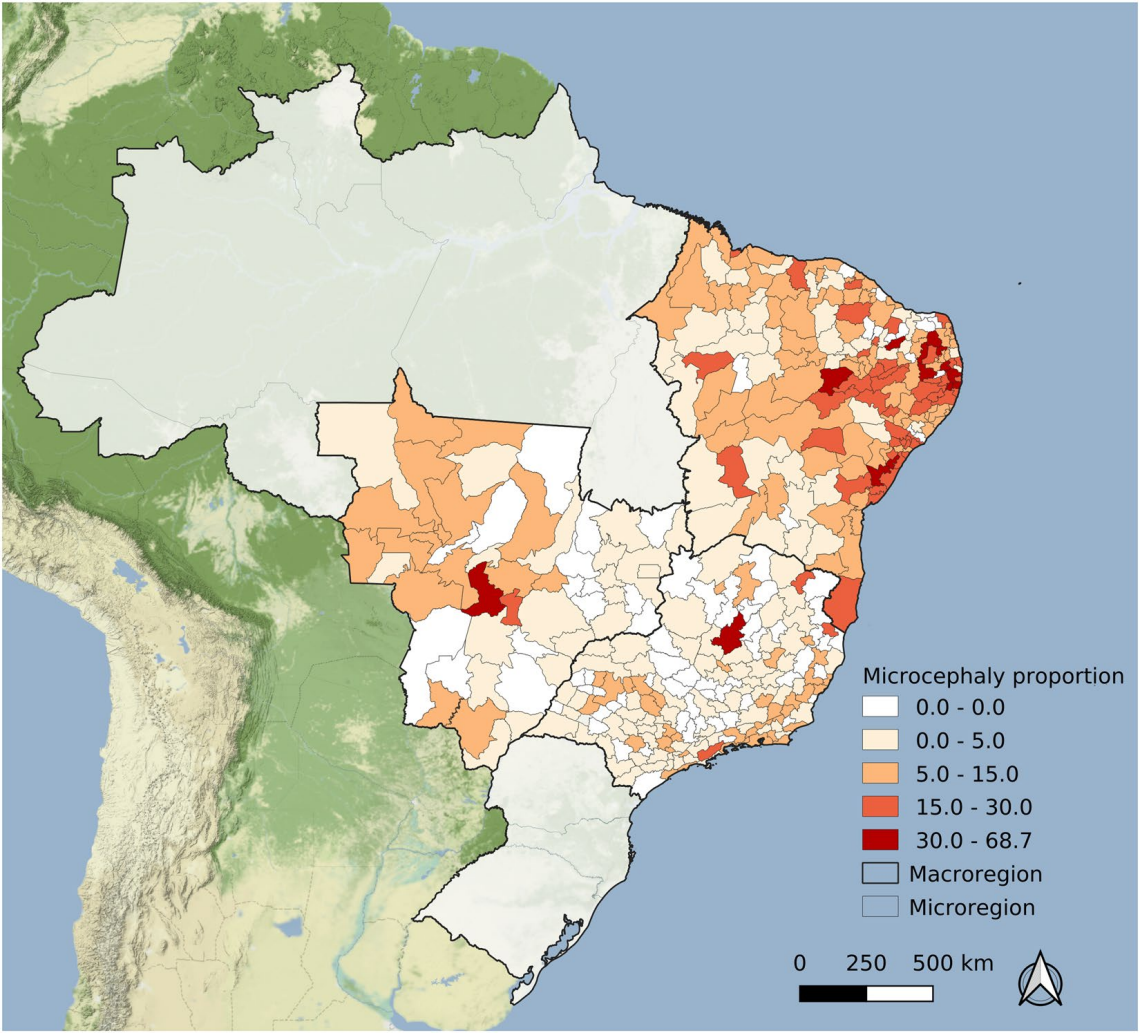
Rate of microcephaly cases per 10,000 live-born infants per microregion, Brazil, 2015–2016. Map created using QGIS version 3.6.3 (QGIS Development Team, 2019, QGIS Geographic Information System. Open Source Geospatial Foundation Project. http://qgis.osgeo.org). Map tiles by Stamen Design, under CC BY 3.0. (https://creativecommons.org/licenses/by/3.0/) Data by OpenStreetMap, under CC BY SA.

The microcephaly epidemic data map (Fig. 3) depicted a different pattern: more than 60% of the areas in the SE and CW regions did not present any cases, while in the NE this proportion was just 20% (Table 1).

**Figure 4:**
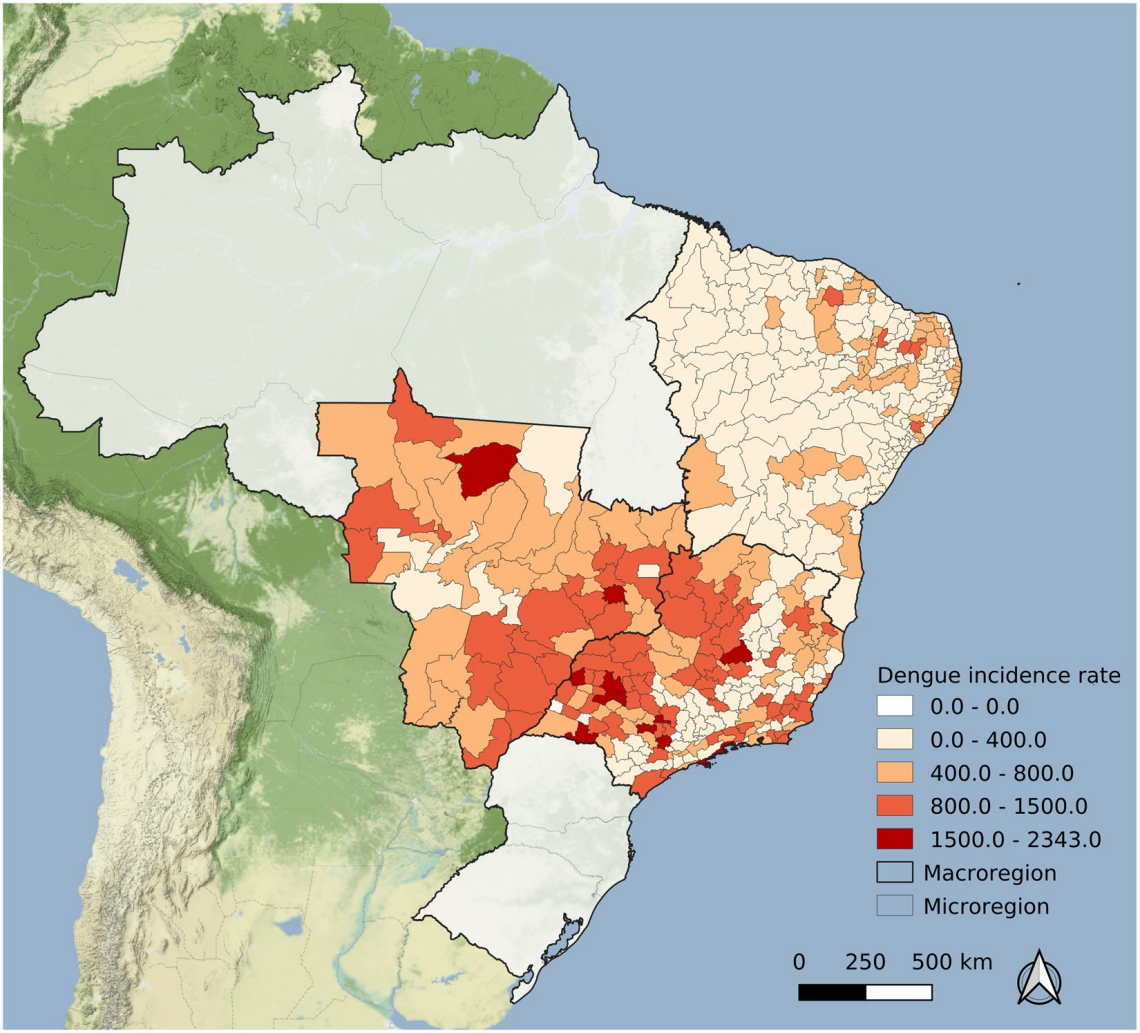
Average annual dengue fever incidence rate per 100,000 over 2010–2014 by microregion, Brazil. Map created using QGIS version 3.6.3 (QGIS Development Team, 2019. QGIS Geographic Information System. Open Source Geospatial Foundation Project. http://qgis.osgeo.org). Map tiles by Stamen Design, under CC BY 3.0. (https://creativecommons.org/licenses/by/3.0/) Data by OpenStreetMap, under CC BY SA.

Figure 4 depicts the mean dengue fever incidence rate over five years (2010–2014). The spatial pattern, as compared with Fig. 3, is inverted: in areas with high microcephaly rates, dengue fever incidence rates were smaller.

**Figure 5:**
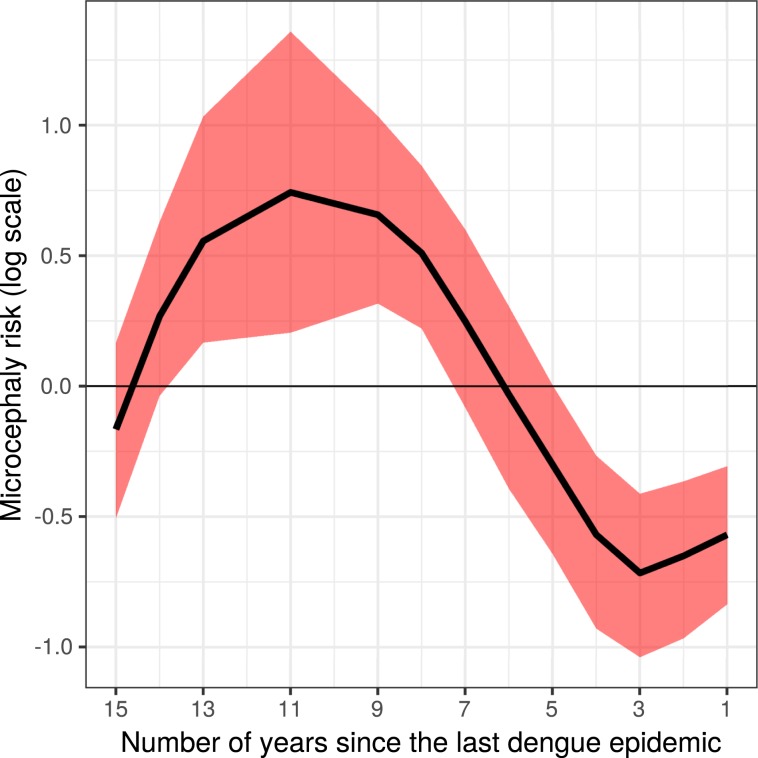
Estimated microcephaly risk (log) and credibility interval by number of years since the last dengue fever epidemic (incidence >400/100,000).

To estimate the association between both indicators – microcephaly rate and previous dengue fever incidence – we estimated the effect of the time lag between the most recent large dengue epidemic and the microcephaly epidemic. Figure 5 presents the main results of this article: the credibility time interval since the last dengue fever epidemic above 400 cases/100,000 inhabitants over the rate of microcephalic neonates. This finding suggests a protective effect up to 6 years prior to an increased risk from 7 to 12 years.

**Figure 6:**
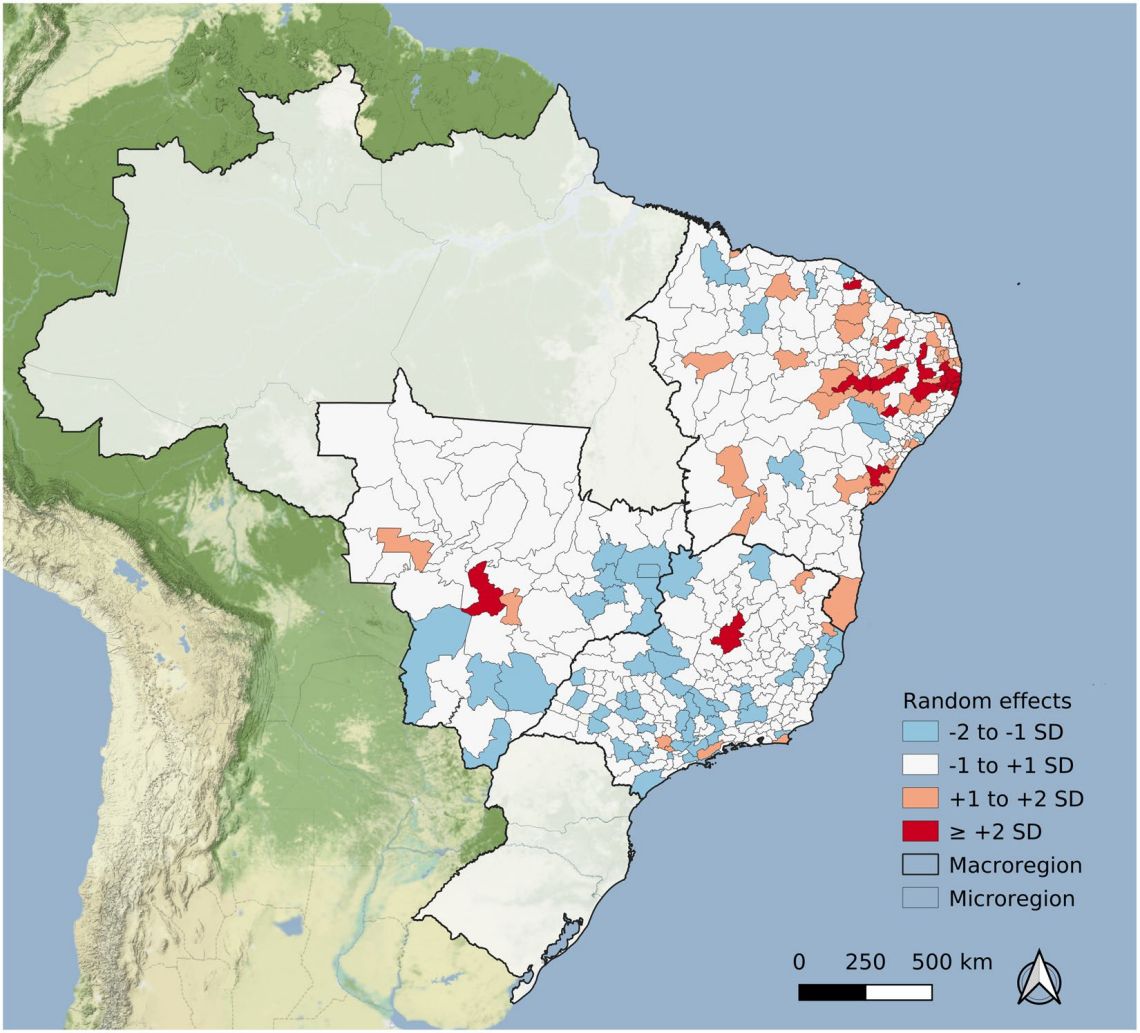
Microregion random effects estimated by the fitted model, presented in standard deviation units. Map created using QGIS version 3.6.3 (QGIS Development Team, 2019. QGIS Geographic Information System. Open Source Geospatial Foundation Project. http://qgis.osgeo.org). Map tiles by Stamen Design, under CC BY 3.0. (https://creativecommons.org/licenses/by/3.0/) Data by OpenStreetMap, under CC BY SA.

The random effect estimated for each microregion, as presented in Fig. 6, suggests that the dengue fever epidemic effect is not enough to explain the large number of microcephaly cases in some areas in the NE. The Supplemental material presents the results of different models, exploring dengue epidemic definitions from 100 to 600/100,000 inhabitants and other statistical distributions. Blue areas are those with a lower rate of microcephaly cases than predicted by the time lag after the last dengue fever epidemic, while orange/red areas are the opposite. In some areas, the estimated random effect is above two standard deviations, mostly spatially concentrated in the NE region.

## Discussion

This is the first article, to the best of our knowledge, in which an association, at the ecological level, between previous dengue fever epidemics and the Zika-related microcephaly epidemic was described. It should be noted that the notification system for Zika was only implemented in February 2016, when the Zika epidemic was decreasing in the NE region. Therefore, we could not control for Zika incidence in the models. It is possible that the differences in microcephaly rates were a consequence of different ZIKV attack rates across the regions, as suggested in a recent paper^37^. In this study, after testing for other concurrent factors, ZIKV was assumed to be the only cause of the microcephaly epidemic, with no unmeasured confounders or effect modifiers. However, if Zika incidence alone explains the excess microcephaly rates, then microcephaly would simply be a proxy for Zika, and it remains unclear as to why the NE region would have presented with a Zika incidence more than ten times larger than anywhere else in Brazil^37^.

**Figure 7:**
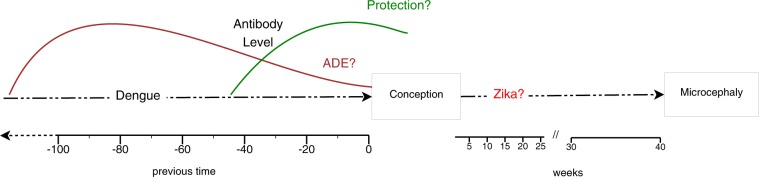
Hypothesized interaction between dengue and Zika infections on the development of microcephaly.

Our hypothesis considers that the antibody decay due to the time interval between infections changes the role of anti-DENV antibodies on ZIKV infection, either to protect or to increase the risk (possibly via antibody-dependent enhancement – ADE), as illustrated in Fig. 7. This hypothesis is based on what is described for sequential heterotypic DENV infections (there are four DENV serotypes). It has been established that a narrow range of pre-existing anti-DENV antibody titres is associated with the risk of severe dengue disease, while high titres protect against clinical illness^29,38^. As the antibody titres decay over time, there is a window of cross-protection between the DENV serotypes. A previous study found that a shorter interval between first and second infections was associated with protection from clinical illness^39^. Additionally to antibodies, DENV-specific CD8^+^ T cells from previous exposure also seems to play a role against ZIKV. Mice primed with DENV and 30 days later challenged with ZIKV during pregnancy presented reduced burden in maternal and fetal tissues, and increased fetal viability compared to non-immune mice. DENV-immune CD8^+^ T cells were required for this cross-protection. However, mice challenged 80 days after DENV priming were not protected, indicating the transient nature of cross-protection again^40^.

Most research on the effect of previous DENV-immunity on the severity of clinical symptoms of ZIKV infection is based on experimental models^41,42^. Cohort studies are the golden standard to epidemiological designs to estimate disease incidence and would give the most reliable understanding of the role of previous DENV exposure on ZIKV infection. However, the reduction in the incidence of Zika after the last epidemic in Brazil in 2016 limits the ability to use prospective studies^43–45^. Some cohorts previously designed to study dengue and that have been adapted to also study Zika are of great value. For example, a recent study in the Pau da Lima cohort in Salvador, a large city of the NE region, Brazil, found that the titres of DENV-antibodies measured before the Zika epidemic were inversely associated with the risk of ZIKV infection^46^. In a paediatric cohort in Nicaragua, prior DENV infection was inversely associated with the risk of symptomatic ZIKV infection^47^.

In a cohort of ZIKV-infected pregnant women, previous DENV infection was not associated with disease severity and abnormal birth outcomes^48^. However, the time interval between the infections and the anti-DENV antibody titres were not considered in the analysis. Our findings indicate that a previous dengue fever epidemic may be related to microcephaly incidence and reinforce the idea of a window of cross-protection and a window of increased risk. Our window of protection is wider than detected in a longitudinal analysis of human immune sera, which showed a cross-neutralizing antibody response to ZIKV on DENV-immune subjects 1–3 years after DENV infection^28^. The larger window at the ecological level is possibly an effect of herd immunity and of multiple exposures to DENV that would boost immunity. This is consistent with a case-control study in which the DENV seroprevalence and the mean number of neutralized serotypes (indicative of multiple exposures) were significantly lower among mothers of neonates with CZS^49^.

We applied a powerful modelling strategy using the INLA approach^32^, which allowed us to simultaneously take into account the non-linear behaviour of the time since the last dengue epidemic (Fig. 5) and the excess variation in each area, as discussed above and shown in Fig. 6. The 18 areas highlighted with higher risks than predicted by the model should be further investigated.

In the easternmost area of the NE region there was an excess of two standard deviations over the number of microcephalic neonates predicted by the model (Fig. 6). In this area, the involvement among researchers and clinicians that led to the early discovery of the causal link between Zika and microcephaly may have generated a hyper-awareness of the problem. The more strict head circumference measurements counterbalanced the usual under-reporting^11,50^. It should be noted that the main surveillance system for neonatal congenital malformation (Latin American Collaborative Study of Congenital Malformations – ECLAMC) did not detect the microcephaly epidemic in the early stages^14^, possibly due to the geographical constraints of the sentinel hospitals. The use of a secondary database such as SINASC, despite its accuracy limitations, together with the more specialized ECLAMC could reinforce a surveillance system for neonatal malformation clustered in space and time.

Dengue epidemics in the previous 15 years were used as a proxy of the herd immunity level among women of reproductive age. The choice of the threshold that we considered high enough to either protect or increase microcephaly risk (of 400/100,000 inhabitants) was data-driven, based on the best fit. The threshold of 300 cases/100,000 inhabitants as high incidence areas, as defined by the Brazilian Ministry of Health, is a number defined *ad hoc*, not sustained by any scientific argument in any guideline of the dengue control program published since 2002.

Other possible factors in the pathway between Zika infection and microcephaly have been suggested, the most important of which is the socioeconomic deprivation in the Brazilian NE^16,51^. This is a typical confounding problem, not easily addressed, mainly due to the larger reservoirs of *Aedes aegypti* in poor areas and increased awareness of the microcephaly risk in the upper socioeconomic classes, inducing either postponing pregnancy during epidemic periods or voluntary abortion, not legal in Brazil^52^.

The main limitations of this study, in addition to not controlling for Zika incidence, are well known: accuracy of microcephaly classification and completeness of dengue fever notification. We recognize the potential inaccuracy on the measurement of neonates’ cephalic perimeter. Considering 10% fewer neonates classified as microcephalic in the NE and 10% more in other regions, the difference would be fourfold, instead of five. As for the under notification of dengue fever, we have no reason to believe there was a trend over the years that would affect our results.

The detection of either ADE or cross-protection between DENV and ZIKV is not a trivial task, either in animal models or humans^42^. The increase in congenital malformations is not easily studied, considering a confluence of different risk factors and the large range of time lags between the potentially harmful event and the outcome. We believe that we brought new evidence that will contribute to building the knowledge regarding the relationship between DENV, ZIKV and congenital Zika syndrome.

## Supplementary information

Supplementary information

## Data Availability

The original datasets are publicly available at http://datasus.saude.gov.br, and the data organised for the present analysis at 10.5281/zenodo.3489428.
