# Supplementary material for: Association of past dengue fever epidemics with the risk of Zika microcephaly at the population level in Brazil
Source: Sci Rep. 2020 Feb 4;10:1752. doi: 10.1038/s41598-020-58407-7 (PMC7000767; doi:10.1038/s41598-020-58407-7)
Supplement: Supplementary file 1 — Supplementary information [file 41598_2020_58407_MOESM1_ESM.pdf]

Association of past dengue fever epidemics with the risk of Zika microcephaly at population level in Brazil

Supplementary material

Marilia Sá Carvalho, Laís Picinini Freitas, Oswaldo Gonçalves Cruz, Patrícia Brasil, Leonardo Soares Bastos

In this supplementary material, we provide the reproducible code to the manuscript, exploratory analysis and outputs.

Required packages

```
library(tidyverse)
library(lubridate)
library(INLA)
```

The data for modelling

```
dengue.df <- read_csv(
  file = "https://zenodo.org/record/3489428/files/dengueBR2001-2014.csv"
  )

micro.df <- read_csv(
  file = "https://zenodo.org/record/3489428/files/microBR2014-2016.csv"
  )
```

The file dengueBR2001-2014.csv contains the notified dengue cases by microregion per year. The included variables (and corresponding name) are:

- Microregion code (microreg);
- Year (Ano);
- Population (Pop);
- Number of notified dengue cases (counts);
- Dengue annual incidence per 100k inhab (DengueTx);

The file microBR2014-2016.csv contains the notified CZS cases in tree-year period from January 2014 to December 2016. The included variables (and corresponding name) are:

- Microregion code (microcod2);
- Number of notified CZS cases (Micro);
- Total of live-births (Nasc);

Now we have to define a "big" dengue epidemic for a given year and microregion, this is done by defining a threshold for the annual dengue incidence. For instance, lets assume that this threshold is 400 cases per 100,000 inhabitants. Hence that combined data frame is built by the following R code:

```
# Big dengue epidemic threshold
BIGEPI <- 400 # 100, 200, 300, 400, 500, 600

dengue.df2 <- dengue.df %>%
  # Creating auxiliary variables
  mutate(EpiHigh = ifelse(DengueTx > BIGEPI,1,0),
         Aux = EpiHigh * Ano) %>%
  # Grouping by microregion
  group_by(microreg) %>%
  summarise(
    # Counting the number of big dengue epidemics
    Xr = sum(EpiHigh, na.rm = T),
    # Defining when the last epidemic occur
    LastEpi = max(Aux, na.rm = T)) %>%
  # If there is no big epidemic we set it as 2000
  mutate(
    LastEpi = ifelse(LastEpi == 0, 2000, LastEpi),
    microcod2 = microreg,
    # Time since the last big dengue epidemic
    Zr = 2015 - LastEpi )

# Joining dengue and CZS dataframes
dengueCZS <- micro.df %>%
  left_join(dengue.df2, by = "microcod2")
```

Model formulation

Let Y_r be the number of notified CZS of microregion r from 2015 and 2016, N_r be the total of live-births in the same period, X_r be the number of "big" dengue epidemics since 2000, and Z_r be the time in years since the last "big" dengue epidemic.

The proposed model is the zero-inflated Poisson model defined as

$$Y_r \sim ZIP(\lambda_r, \pi),$$

where π is a hyper-parameter defined as the probability of zero coming from a degenerated distribution, hence $1 - p$ is the probability of zero coming form a Poisson. The parameter λ_r

is defined as

$$\log(\lambda_r) = \log(N_r) + \alpha + \beta X_r + \gamma(Z_r) + \delta_r.$$

The coeffients α and β are fixed effects, $\gamma(Z_r)$ is time-varying second-order random walk to emulate a spline-like function of Z_r, i.e. $\gamma(z) - 2\gamma(z-1) + \gamma(z-2) \sim N(0, \tau_\gamma)$, and δ_r is a microregion Gaussian random effect, i.e. $\delta_r \sim N(0, \tau_\delta)$.

Vague priors were defined for all hyper-parameters.

```
model.equation <- Micro ~ 1 + Xr +
  # Microregion effect
  f(microcod2, model = "iid") +
  # Time since last epidemic effect
  f(Zr, model = "rw2")

likelihood.family <- "zeroinflated.poisson.1"
# Other tested likelihoods
# "poisson", "nbinomial", "zeroinflated.nbinomial.1"

output <- inla(formula = model.equation,
               data = dengueCZS,
               family = likelihood.family,
               E = Nasc / 10000,
               control.compute = list(waic=TRUE) )
```

Results

The results bellow are for the zero inflated Poisson likelihood and the threshold for big dengue epidemic set as 400 dengue cases per 100 thousand inhabitans.

Fixed effects (α and β)

```
output$summary.fixed[,c(1,3,5)]
```

```
##                   mean 0.025quant 0.975quant
## (Intercept) 2.15297538 1.91727558  2.3845072
## Xr          0.07423508 0.01912741  0.1296944
```

Hyperparameters (π, τ_γ, τ_δ)

```
output$summary.hyperpar[,c(1,3,5)]
```

```
##                                                                    mean
```

```
## zero-probability parameter for zero-inflated poisson_1  0.00581059
## Precision for microcod2                                  1.28351014
## Precision for Zr                                        57.37709006
##                                                          0.025quant
## zero-probability parameter for zero-inflated poisson_1 0.0007110325
## Precision for microcod2                                1.0142237361
## Precision for Zr                                       9.5138896164
##                                                        0.975quant
## zero-probability parameter for zero-inflated poisson_1   0.019730
## Precision for microcod2                                  1.578808
## Precision for Zr                                       211.938835
```

Random effect $\gamma(Z_r)$

Effect of time since the last big dengue epidemic.

```
aux.plot <- output$summary.random$Zr

p <- ggplot(aux.plot, aes(x = ID, y = `0.5quant`,
                          ymax = `0.975quant`,
                          ymin = `0.025quant`)) +
  #geom_ribbon( fill = "gray") +
  geom_ribbon( alpha = .5, fill = "red") +
  geom_line(lwd = 2) +
  geom_hline(yintercept = 0) +
  theme_bw(base_size = 18) +
    xlab("Number of years since the last dengue epidemic") +
#  xlab("Number of years since the last largest dengue epidemic") +
  ylab("Microcephaly risk (log scale)") +
  scale_x_continuous(breaks = seq(1,15, by=2), trans = "reverse" )
```

Effect of time since the last big dengue epidemic.

```
ggplot(aux.plot, aes(x = ID, y = `0.5quant`,
                          ymax = `0.975quant`,
                          ymin = `0.025quant`)) +
  #geom_ribbon( fill = "gray") +
  geom_ribbon( alpha = .5, fill = "red") +
  geom_line(lwd = 2) +
  geom_hline(yintercept = 0) +
  theme_bw(base_size = 18) +
  xlab("Year of the last big dengue outbreak") +
  ylab("Microcephaly risk (log scale)") +
  scale_x_continuous(breaks = seq(1,15, by=2), labels = seq(2014,2000, by=-2), trans =
```

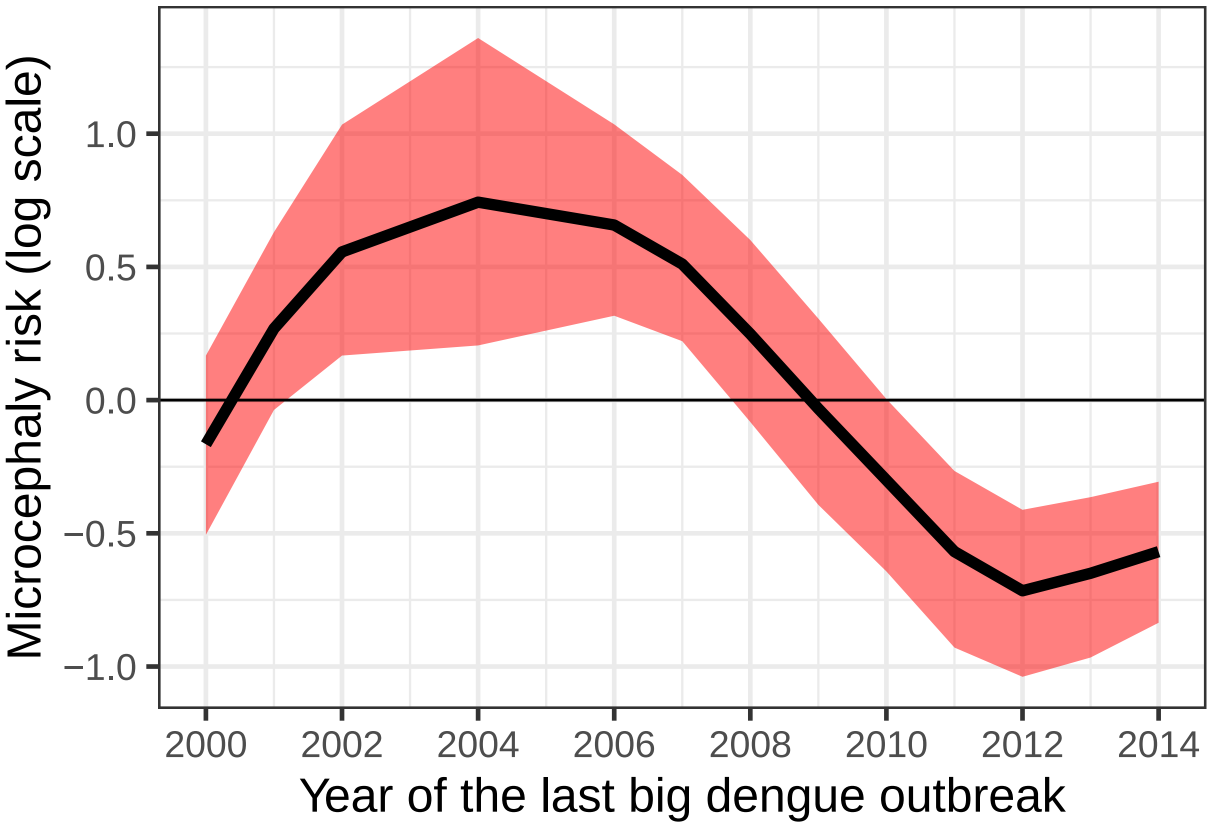

Random effect δ_r

Microregion random effect. The microregion region code is the official code of the Brazilian Institute of Statistics and Geography (IBGE), so the random effects could be spatially represented in a map. In the plot bellow the data order is arbitrary however the colors represent different macroregions, 2 for NE region, 3 for SE, and 5 for CW.

```
aux2.plot <- output$summary.random$microcod2[,c(1,5,4,6)]
aux2.plot$macroregion <- substr(aux2.plot$ID, start = 1, stop = 1)

ggplot(aux2.plot,
             aes(x = as.character(ID),
                 y = `0.5quant`,
                 ymax = `0.975quant`,
                 ymin = `0.025quant`,
                 color = macroregion)) +
  geom_point() + geom_errorbar() +
  geom_hline(yintercept = 0) +
  theme_bw(base_size = 18) +
  ylab("Microcephaly risk (log scale)") +
  xlab("Microregion") +
  theme(axis.text.x=element_blank(),
```

```
        axis.ticks.x=element_blank() )
```

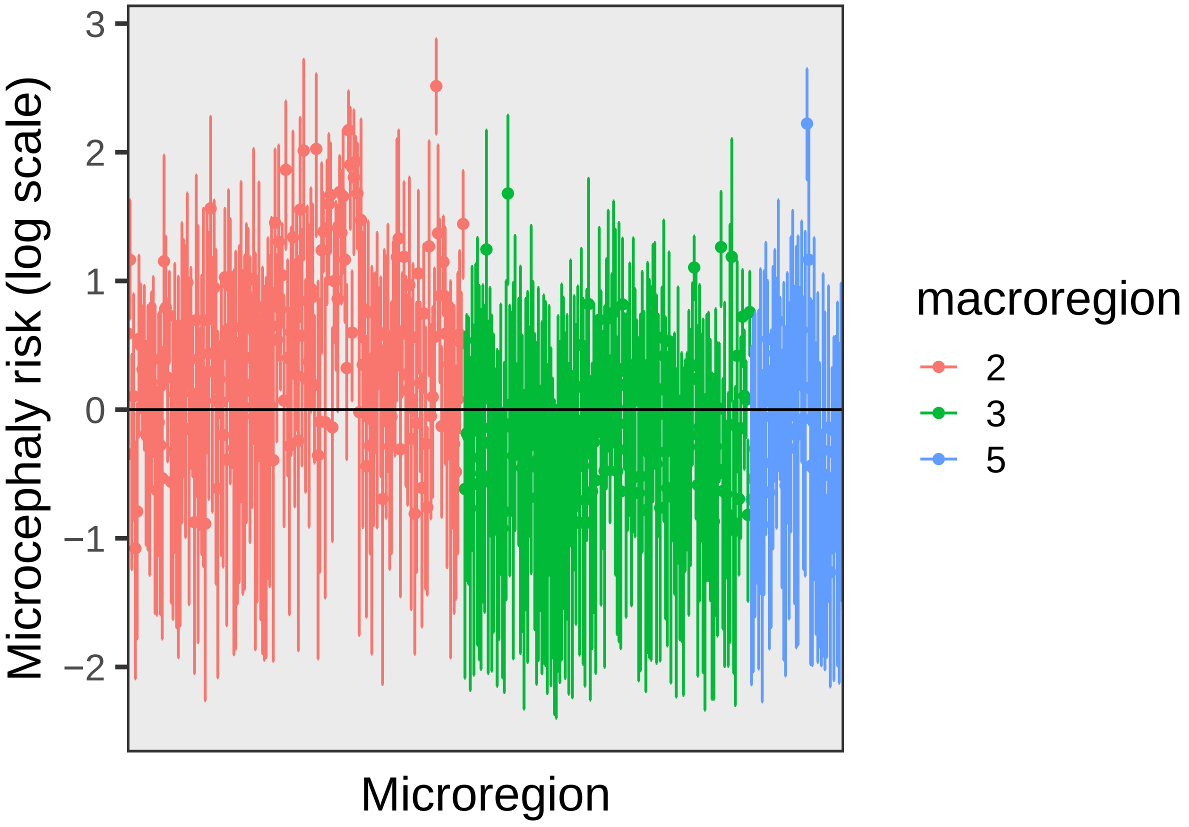

WAIC table of all tested models

The table below presents the WAIC for all tested models by varying the likelihood (Poisson, Negative binomial, Zero-inflated Poisson, and Zero-inflated negative binomial) and the epidemic threshold for defining a big dengue epidemic, values varying from 100 to 600. You can notice that the ZIP model with a threshold of 400 cases per 100 thousand inhabitants is the best one according to this criterion, the smaller the better.

Likelihood	100	200	300	400	500	600
Pois	1712.22	1713.75	1710.00	1705.93	1706.86	1710.32
Nbin	2063.60	2076.43	2066.50	1766.01	2067.50	2062.14
ZIP	1710.27	1713.22	1708.27	1704.39	1708.24	21232.14
ZINB	2065.74	2079.14	2070.58	1783.26	1797.69	2009.46

Table 1: Watanabe-Akaike information criterion for different models varying the likelihood (rows) and the big dengue epidemic threshold (columns).

You may also notice a very large value for WAIC for the ZIP model with a threshold of 600, this occur because the model didn't fit properly due to numerical instability problems.
